# Data on the optimizations of expression and purification of human BiP/GRP78 protein in *Escherichia coli*

**DOI:** 10.1016/j.dib.2016.08.006

**Published:** 2016-08-09

**Authors:** Jiao Yang, Lei Zhou, Qinglian Liu

**Affiliations:** Department of Physiology and Biophysics, School of Medicine, Virginia Commonwealth University, VA 23298, USA

**Keywords:** Hsp70, BiP/GRP78, Protein expression and purification

## Abstract

Human BiP/GRP78 is involved in the folding and assembly of proteins in the endoplasmic reticulum. The proteins for crystallization in good amount and quality are prerequisites for obtaining ideal crystals. To meet these requirements, different BiP/GRP78 constructs, competent cells, vectors, and concentrations of inducer were tested in order to obtain soluble BiP/GRP78 protein with the highest amount and best purity. The BiP–T229A–L_3,4′_–Smt3 fusion protein was expressed in a soluble manner and finally purified with the highest purity using size exclusion chromatography, which was suitable for further protein crystallization.

**Specifications Table**

**Table:** 

Subject area	Molecular Biology
More specific subject area	Protein expression and purification
Type of data	Pictures. Images of gel electrophoresis.
How data was acquired	SDS-PAGE electrophoresis and FPLC
Data format	Analyzed
Experimental factors	BiP(WT, T229A, and T229A–L_3,4′_)–Smt3 fusion protein. IPTG concentration during protein expression. Affinity chromatography, ion exchange chromatography and size exclusion chromatography
Experimental features	Optimization on protein expression and purification
Data source location	Virginia Commonwealth University, Richmond, VA, USA
Data accessibility	Data is within this article

**Value of the data**

**Table:** 

• The data indicates that the production of full-length recombinant proteins in the bacterium E. *coli* can be improved by varying the constructs and concentration of inducer during protein expression. • The data indicates that pSMT3 vector may be used to stabilize the full-length recombinant proteins expressed in the bacterium E. *coli*. • The optimized purification procedure may be still good for the expression of general eukaryotic Hsp70s in the bacterium E. *coli*.

## 1 Data

**Fig. 1:**
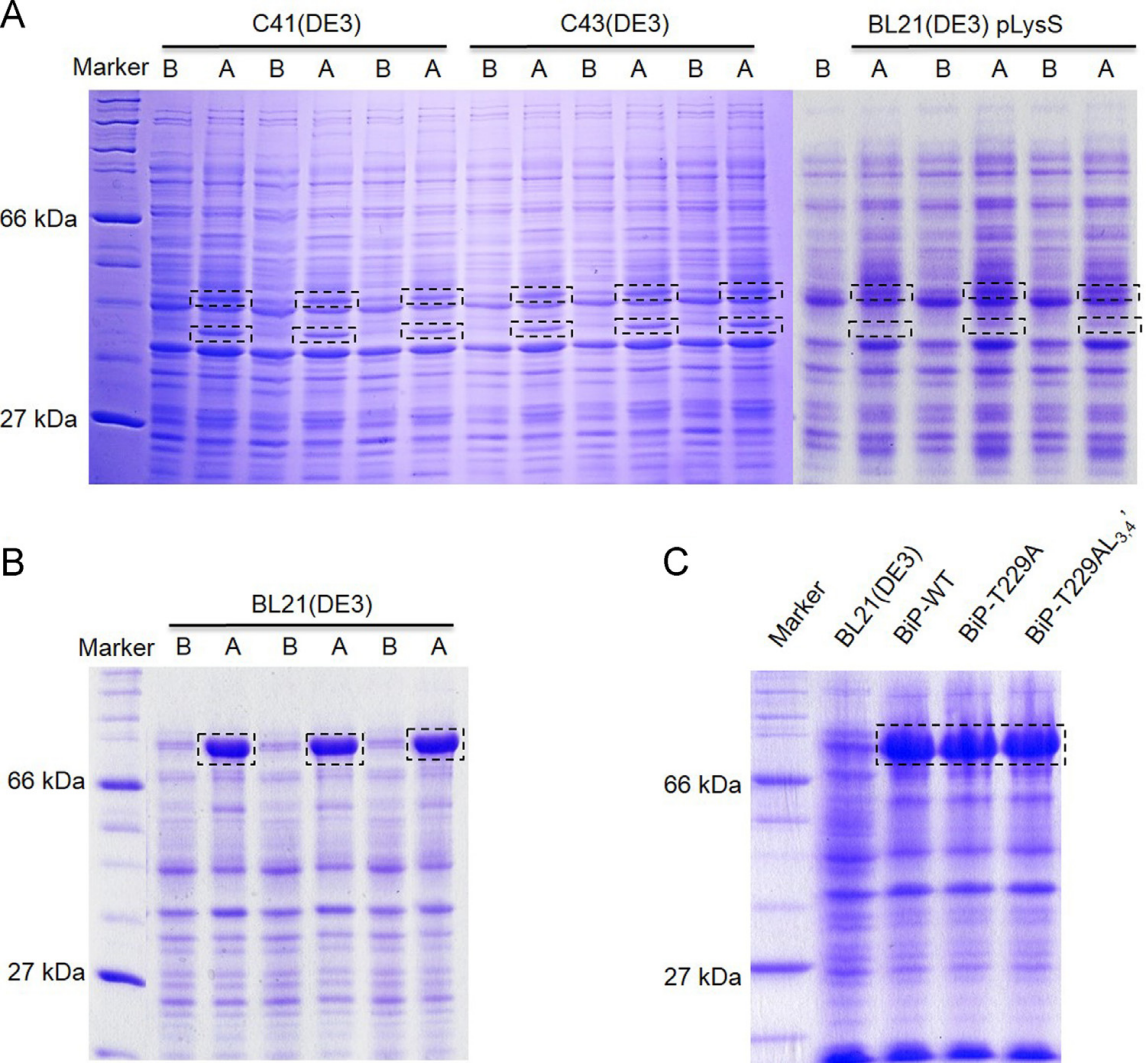
10% SDS-PAGE analysis of small-scale expression for the human BiP constructs with or without Smt3 tag at 30 °C using different concentrations of IPTG. (A) The recombinant full-length human BiP constructs without Smt3 tag could not be expressed in *E. coli* C41(DE3), C43(DE3), or BL21(DE3) pLysS. Lane 1: protein marker; lane 2–7 were protein samples from *E. coli* C41(DE3), lane 8–13 were protein samples from *E. coli* C43(DE3), Lane 14–19 were protein samples from *E. coli* BL21(DE3)pLysS. Lane 2,4,6,8,10,12,14,16, and 18 represented protein samples before IPTG-induction; lane 3, 9, and 15 were protein samples after induction using 0.25 mM IPTG; lane 5, 11, and 17 were protein samples after induction using 0.5 mM IPTG; lane 7, 13, and 19 were protein samples after induction using 1 mM IPTG. (B) Recombinant BiP–WT–smt3 fusion protein was expressed in *E. coli* BL21(DE3). Lane 1: protein marker; lane 2, 4, and 6 represented protein samples before induction, lane 3, 5, and 7 represented protein samples after induction using 0.25, 0.5, and 1 mM IPTG, respectively. (C) The expression of BiP–WT–smt3, BiP–T229A–Smt3, and BiP–T229A–L_3,4′_–Smt3 fusion proteins in *E. coli* BL21(DE3). Lane 1: protein marker; lane 2: negative control (NC); lane 3: expressed BiP–WT–smt3; lane4: expressed BiP–T229A–Smt3; lane 5: expressed BiP–T229A–L3,4′–Smt3.

**Fig. 2:**
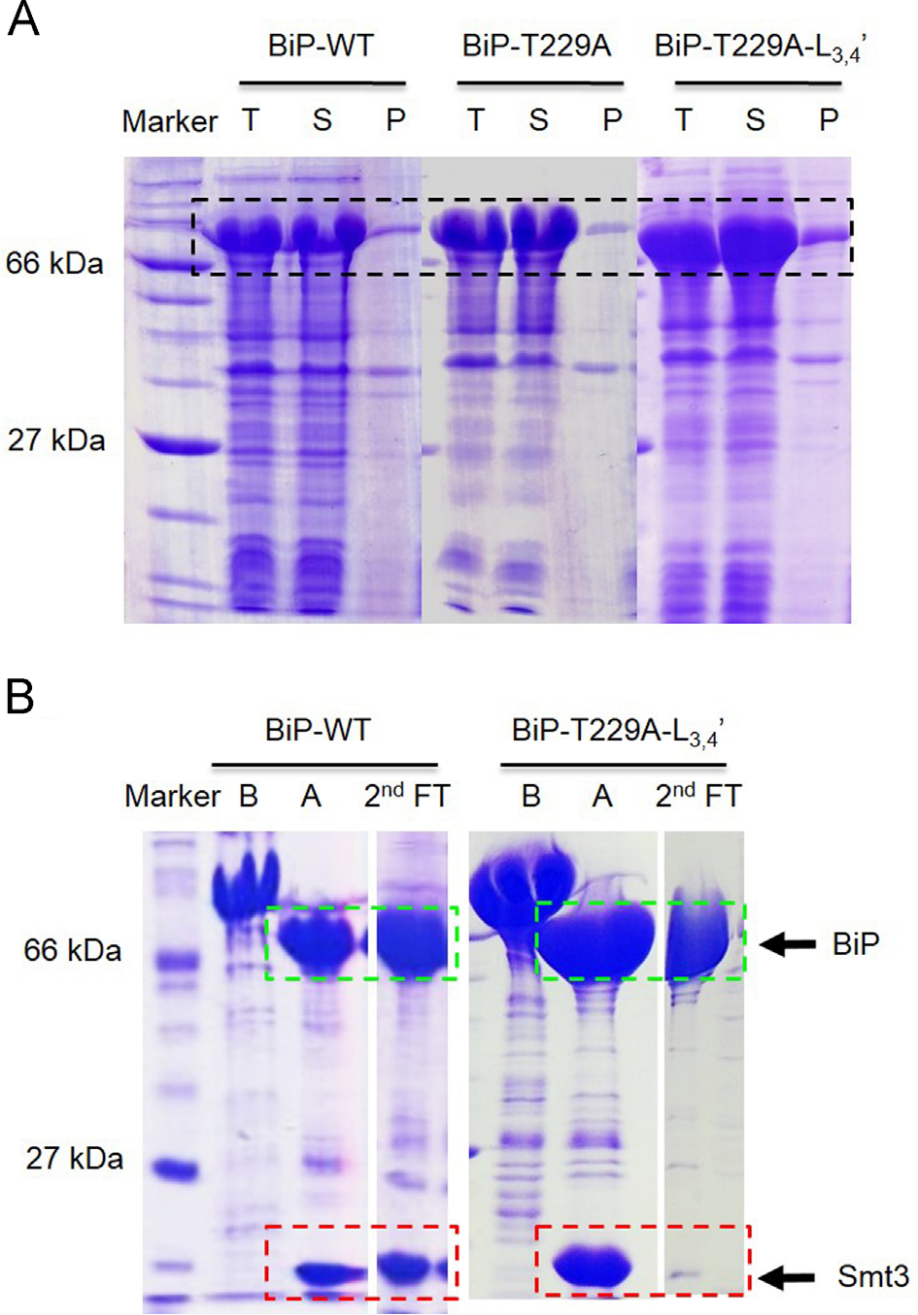
10% SDS-PAGE analysis of the solubility and purity of BiP proteins produced from small-scale expressions at 30 °C for the BiP–WT, BiP–T229A, and BiP–T229A–L_3,4′_ constructs tagged with Smt3. (A) The solubility test for each BiP–Smt3 protein. Expression of BiP–WT–Smt3 (lane 2–4), BiP–T229A–Smt3 (lane 5–7), and BiP–T229A–L_3,4′_–Smt3 (lane 8–10) at 30 °C were shown on each gel which respectively represented total protein sample (lane 2, 5, 8), supernatant protein (lane 3, 6, 9) and the protein sample from pellet (lane 4, 7, 10). (B) The purification result after running 2nd Histrap column. Lane 1: protein marker; lane 2: BiP–WT–Smt3 protein samples before digestion by Ulp1; lane 3: BiP–WT–Smt3 protein samples after digestion by Ulp1; lane 4: purified BiP–WT protein samples after 2nd Histrap column ; lane 5: BiP–T229A–L_3,4′_–Smt3 protein samples before digestion by Ulp1; Lane 6: BiP–T229A–L_3,4′_–Smt3 protein samples after digestion by Ulp1; lane 7: purified BiP–T229A–L_3,4′_ protein samples after 2nd Histrap column. The BiP protein bands were marked in green box and the Smt3 protein bands were marked in red box.

**Fig. 3:**
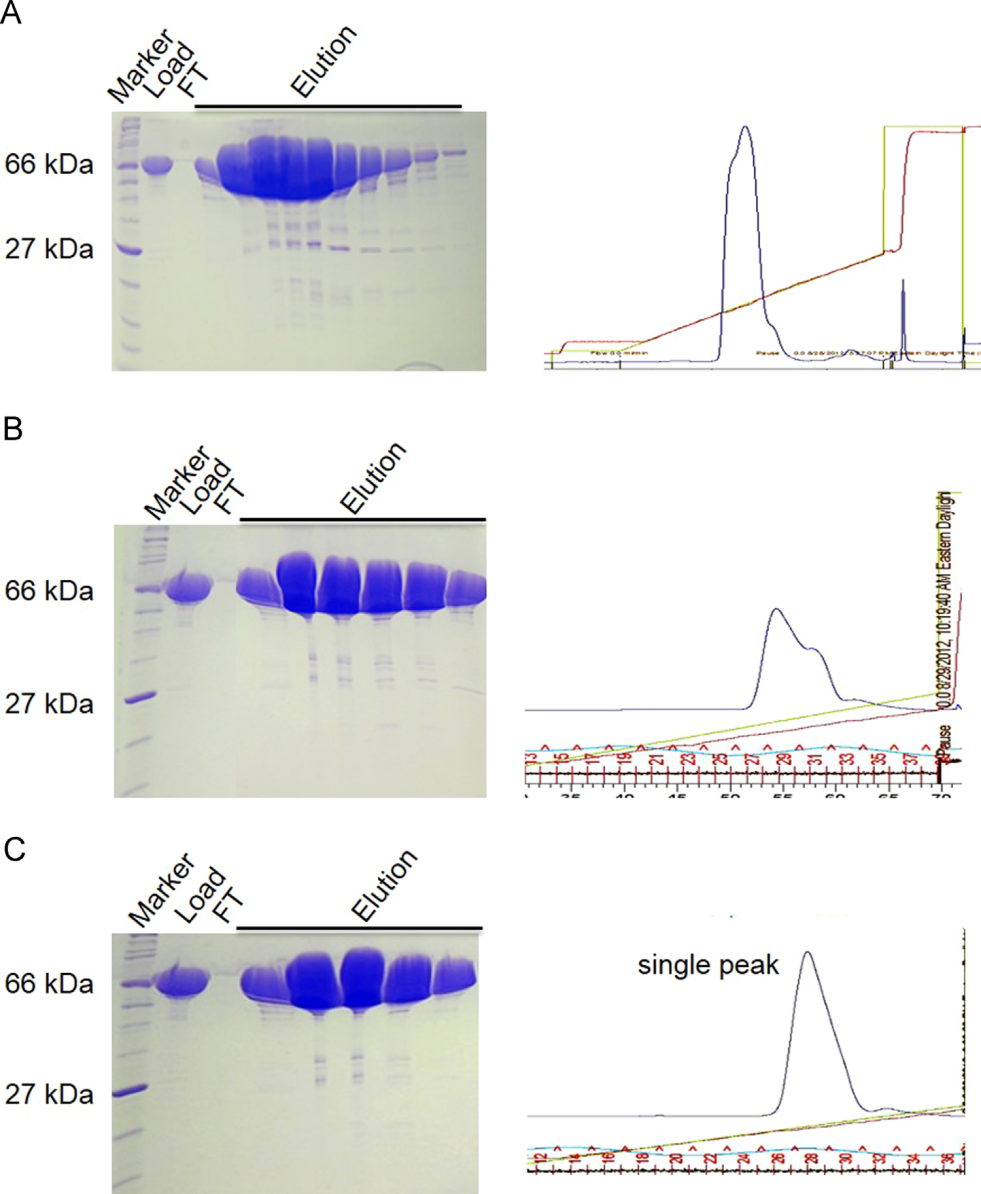
10% SDS-PAGE analysis and FPLC results of the purification of BiP–T229A–L_3,4′_ with Hitrap Q column. (A) purified BiP–T229A–L_3,4′_ on the first HiTrap Q column; (B) purified BiP–T229A–L_3,4′_ on the second HiTrap Q column; (C) purified BiP–T229A–L_3,4′_ on the third HiTrap Q column. The pictures on the right were profiles of FPLC results.

**Fig. 4:**
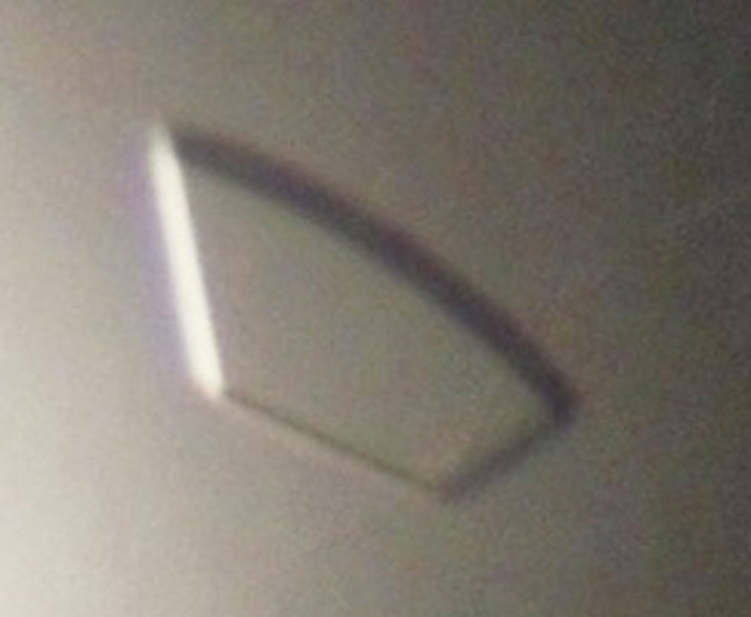
Crystal image of BiP–T229A–L_3,4′_.

Fig. 1 shows the SDS-PAGE analysis comparing the induction of the human BiP protein expression tagged with or without Smt3 tag at different conditions. Fig. 2A shows the solubility of the WT, T229A, and T229A-L_3,4′_ BiP–Smt3 fusion proteins. Fig. 2B shows the purifications of BiP proteins (after digestion by Ulp1) on HiTrap Q column, the WT or T229A BiP proteins could not be separated from Smt3, but the T229A–L_3,4′_–BiP protein could be completely separated from Smt3. Fig. 3 shows the FPLC result and SDS-PAGE analysis for the purified BiP–T229A–L_3,4′_ protein on HiTrap Q column. Fig. 4 shows the crystal picture of BiP–T229A–L_3,4_ protein.

## 2 Experimental design, materials and methods

### 2.1 The constructs for protein expression

Standard procedure was followed for the construct design according to the description of pET vector cloning strategies [1]. To express BiP protein, the pET-28–BiP construct was designed as below. The BiP (25–630) coding DNA sequence was double digested by *NcoI* and *XhoI* restriction enzymes (NEB) in reaction buffer 4, after purified with Gel extraction kit (Qiagen), it was inserted into a predigested (*NcoI*/*XhoI*) pET-28(a+) vector (Kan^R^) carrying the N-terminal His tag by following the manual of In-fusion (HD EcoDry) cloning kit (Clontech, Takara Bio Company).

To express the BiP–Smt3 fusion protein, pSMT3–BiP construct was designed similarly but using pSMT3 vector (a generous gift from Dr. Chris Lima, Sloan-Kettering Institute). In brief, after double-digesting pSMT3 vector by *BamHI* and *XhoI*, BiP (25–630) coding DNA sequence was inserted into pSMT3 vector between these two sites by using the In-fusion (HD EcoDry) cloning kit (Clontech, Takara Bio Company). *BamHI* recognition sequence (GGATCC) connected Smt3 and BiP coding sequence, which would be translated to Gly and Ser and served as the Ulp1 cleavage site to separate BiP from Smt3 protein.

### 2.2 Protein expression and purification

The competent cell preparation for all different strains including BL21(DE3), BL21(DE3) pLysS, C41(DE3), and C43(DE3) were essentially the same as described by Agilent Technologies Company and Lucigen Corporation. The pET-28–BiP or pSMT3–BiP DNA was transformed using calcium chloride protocol as previously described [2]. The following day, pick up a single colony and inoculate into 10 ml LB broth containing 25 µg/ml kanamycin or LB broth containing both 25 µg/ml kanamycin and 25 µg/ml chloramphenicol (for BL21(DE3) pLysS transformation). The culture was incubated at 37 °C overnight with vigorous shaking, and 1:100 diluted to a fresh medium the next day. When cellular density reached OD_600_=0.6–0.8, culture would be induced by the addition of IPTG at different concentration (0.25 mM, 0.5 mM, and 1 mM) and incubated at 30 °C for another 5–6 h with shaking. 1 ml cell culture was finally collected and resuspended in SDS loading buffer (100 µl per 1 OD_600_) for SDS-PAGE analysis.

The recombinant BiP–Smt3 proteins (WT, T229A, and T229A–L_3,4′_) were purified similarly as mentioned for DnaK–Smt3 protein [3]. First, the fusion proteins passed through a HisTrap column with the buffer containing 2×PBS, after removing Smt3 tag by UlP1 protease, the BiP protein was further separated from the cut Smt3 tag on a second HisTrap column. The single phase BiP–T229A–L_3,4′_ protein was acquired by using HiTrap Q column with 25 mM Hepes (pH=7.5) as the buffer. It took several rounds to get the pure BiP–T229A–L_3,4′_ protein in single phase, so the incubation of BiP protein with ATP helped to reach this goal. Finally, the BiP protein was further purified on a Superdex 200 16/60 column and concentrated up to ~30 mg/ml in buffer A (5 mM Hepes-KOH, pH7.5 and 10 mM KCl). All BiP proteins were flash frozen in the liquid nitrogen before storing at −80 °C freezer.
